# Do Public Perceptions of the Environment Align With Empirical Measures?

**DOI:** 10.1002/snz2.70027

**Published:** 2026-02-19

**Authors:** Pamela L. Booth, Lynette J. McLeod, Philip Stahlmann‐Brown

**Affiliations:** ^1^ Bioeconomy Science Institute Wellington New Zealand; ^2^ School of Psychology Speech and Hearing University of Canterbury Christchurch New Zealand

**Keywords:** empirical measures, environmental management, New Zealand, perceptual biases, public perceptions

## Abstract

To create effective environmental communications and policies, it is crucial to understand how laypeople, scientists, and experts perceive the environment. This study, based on a large‐scale, nationally representative survey from Aotearoa New Zealand and scientific reports and literature, examines the similarities and differences in perceptions across ten environmental domains: air; protected natural areas; native bush and forests; marine environments; coastal waters and beaches; marine plants and animals; terrestrial (land and freshwater) plants and animals; natural environments in towns and cities; wetlands; and rivers and lakes. The New Zealand public rated their air quality highest and rivers and lakes lowest, aligning with empirical measures and expert opinions. For other domains, public perceptions varied, often seeing the overall state as better than what the scientific studies indicated. These discrepancies highlight the complex factors influencing perceptions, such as public awareness, visibility, familiarity with environmental issues, and media influence. By highlighting these similarities and differences, we aim to help laypeople, scientists, and policymakers recognise their perceptual biases, improve communication of scientific findings, and assist in creating regulations that involve the entire community in environmental improvement.

## 1 Introduction

The United Nations Environmental Programme's “Global Environment Outlook” paints a grim picture of the state of the environment (United Nations Environmental Programme 2019): We are on a brink of a sixth mass extinction, halting or reversing environmental degradation has been slow to progress, and pollution is recognised as the single largest risk to human health worldwide (Landrigan et al. 2018; United Nations Environmental Programme 2019). Beliefs that the environment will always provide, that development is a zero‐sum game with the environment, and that technology will boundlessly improve natural resource efficiency influence both how individuals interact with the environment and how decision makers manage the environment (United Nations Environmental Programme 2019). However, if beliefs are based on perceptions of ecological states that differ from scientific knowledge, the behaviours, policies, and regulations on which these beliefs rely may perpetuate environmental degradation (Bennett 2016).

To make better environmental policy and improve communications, we need to understand the similarities and differences in how laypeople, scientists, and experts view the condition of the environment around them. In this paper, we review the literature on the alignment of perceptions of ecological states and its implications. Then, using Aotearoa New Zealand (hereafter, “New Zealand”) as a case study, we synthesise where these similarities and differences exist within and across ten environmental domains: air; protected natural areas; native bush and forests; marine environments; coastal waters and beaches; marine plants and animals; terrestrial (land and freshwater) plants and animals; natural environments in towns and cities; wetlands; and rivers and lakes. By illustrating and synthesising these similarities and differences, we aim to help laypeople and experts alike recognise their own perceptual biases, thereby opening space for better decision making.

For clarity, we define three key terms used throughout this paper. ‘Alignment’ refers to instances in which public perceptions of environmental conditions correspond with an expert assessment. ‘Misalignment’ occurs when public perceptions diverge from these measures, either by overestimating or underestimating environmental conditions. ‘Empirical consensus’ is the prevailing understanding of environmental conditions based on systematically collected data, scientific literature, and expert interpretation.

We refer to “scientists”, “managers”, and “experts” as distinct but sometimes overlapping groups involved in environmental assessment. Scientists are individuals engaged in systematic research and data collection to understand environmental processes and conditions. Managers are practitioners responsible for implementing environmental policies, conservation strategies, and resource management on the ground. “Experts” is a broader term that may include both scientists and managers as well as individuals with specialised knowledge (e.g., traditional ecological knowledge holders or technical advisors). While these groups may share similar goals, they often approach environmental assessment from different perspectives: scientists focus on empirical evidence and theory, managers on practical application and outcomes, and experts may integrate both scientific and experiential knowledge. Where the terms are used interchangeably, it reflects the collaborative nature of environmental work in New Zealand, but distinctions are made where relevant to the interpretation of empirical consensus or public perception.

### 1.1 Conceptual Framework

**FIGURE 1:**
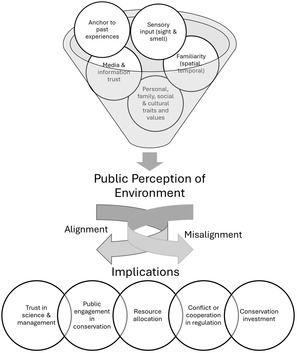
Conceptual framework illustrating the relationships among determinants of perception, public perception of environmental conditions, alignment or misalignment with empirical consensus, and implications for policy and management.

A conceptual framework provides a structured lens through which to examine the complex relationships between public perceptions, the empirical consensus, and their implications for policy and management. This framework helps clarify how perceptions are formed, how they compare to scientific assessments, and what consequences these comparisons have for decision making. It comprises four key components: the determinants that shape public perception, the perceived state of environmental conditions, the degree of alignment or misalignment with empirical consensus, and the resulting implications for environmental policy and management. Figure 1 illustrates these relationships, offering a visual overview of how these elements interact and influence one another.

### 1.2 Public Perceptions of the Environment

A complex interplay of personal, social, and cultural factors influences the lens through which people view the environment (Table 1). Personal characteristics, values, and beliefs, as well as social, cultural, and family norms, can be important factors. For example, higher‐income individuals are less likely to underestimate the quality of freshwater bodies than lower‐income individuals, while older people are more likely to overestimate the quality of freshwater bodies than younger people (Artell et al. 2013). Men are more likely to perceive marine species through a utilitarian lens (e.g. cod are used for food), while women are more likely to perceive marine species through an aesthetic lens (e.g. puffins are visually interesting) in UK waters (Jefferson et al. 2014). In another example, the cultural significance of public green spaces increases the perceived quality and perceived physical size of those places (Aoshima et al. 2018). Similarly, the relational values associated with wetlands may differ across a person's culture, upbringing, value system, and other personal characteristics, for example, wetlands have historical, cultural, and economical importance to Māori (Harmsworth 2013).

**TABLE 1:** Summary of factors found influencing the perceptions across 10 key environmental domains.

Domain	Factor(s)	Reference
Air	Senses (sight); Anchoring (low frequency events)	Liu et al. (2021); Williams and Bird (2003)
Protected natural areas	Familiarity (awareness); Management intensity (e.g., see lots of working being done)	McClenachan et al. (2018); Petrosillo et al. (2007); Rodríguez‐Rodríguez (2012)
Native bush and forests	Social values; Familiarity (awareness, temporal)	Castillo et al. (2021); Domínguez‐Torreiro and Soliño (2011), a
Marine environment	Information (trust); Senses (sight, small)	Jefferson et al. (2014, 2015)
Coastal waters and beaches	Senses (sight, smell); Familiarity (temporal); Anchoring (illness, past experience); Information (trust, media, notices); Aesthetics; Nearby built amenities	Aretano et al. (2017); Hamal et al. (2021); Pendleton et al. (2001); Quilliam et al. (2019); Rodella and Corbau (2020); Pratap et al. (2013)
Marine plants and animals	Gender; Social values; Information (trust)	Jefferson et al. (2014, 2015)
Terrestrial plant and animals	Gender; Social values; Familiarity; Aesthetic preferences	Kiley et al. (2017); Jaun‐Holderegger et al. (2022)
Natural environments in towns and cities	Culture; Senses (sight, smell); Aesthetic preferences	Aoshima et al. (2018); Baranzini et al. (2010); Hofmann et al. (2012); Kothencz and Blaschke (2017); Steinwender et al. (2008)
Wetlands	Senses (sight, smell); Aesthetic preferences	Cottet et al. (2013)
Rivers and lakes	Demographics (age, income); Senses (sight, smell); Familiarity (spatial, temporal); Loss aversion; Anchoring (past experiences); Aesthetic preferences	Ahitiainen et al. (2015); Artell et al. (2013); Kataria et al. (2012);^a^ Liu et al. (2021); Marsh et al. (2011);^a^ Steinwender et al. (2008)

a Study found that a difference between perceptions and empirical measures exists but did not explore the reasons for or factors influencing that difference

Familiarity with the environment is important for aligning perceptions with empirical measures (Petrosillo et al. 2007), especially the degree of familiarity as measured by time (Aretano et al. 2017; Artell et al. 2013) and distance (Aretano et al. 2017; Barkmann et al. 2008). A lack of familiarity leaves space for other factors to influence perceptions, such as media and anchoring, often to the detriment of increasing interactions with the environment or correcting misinformation (e.g. Pendleton et al. 2001). Portrayals of environmental issues in the media have been shown to influence environmental perceptions (Jefferson et al. 2014; Pendleton et al. 2001; Quilliam et al. 2019). For example, people may trust one source of information more than another (Bennett 2016; Kirk et al. 2022; Pratap et al. 2013). Moreover, reporting can lead to misunderstandings about the state of the environment; for example, Santos and Crowder (2021) found that online news media coverage of sea turtles disproportionately focuses on threats that may not be the most important for the conservation of these animals.

Perceptions of the environment can sometimes be anchored to past experiences of illness (Pratap et al. 2013), historical knowledge (Hamel et al. 2021), notable but low‐frequency events (Lui et al. 2021), or information/disinformation from media (Pendleton et al. 2001). For example, Liu et al. (2021) found that people remember hot and hazy days as occurring more frequently than they did and, as a result, had poor perceptions of air quality in comparison with the empirical consensus. Once anchored, it can also be difficult to update beliefs (Kataria et al. 2012; Marsh et al. 2011). In addition, people may anchor their perceptions of what a landscape “should” look like to that with which they grew up (Kaur et al. 2004). For example, Verbrugge and van den Born (2018) found that people who were born in an area were less positive about infrastructure changes to local waterways that improved flood safety, ecological health of rivers, and navigability of rivers than people who moved there later in life.

Sensory input has also been shown to influence perceptions of urban rivers (Steinwender et al. 2008), wetlands (Cottet et al. 2013), urban green spaces (Hofmann et al. 2012; Kothencz and Blaschke 2017), forests (Aoshima et al. 2018), overall environmental quality (Liu et al. 2021), and coastal waters and beaches (Pratap et al. 2013; Rodella and Corbau 2020). For example, strong smells, turbid or murky waters, and floating debris signal inferior quality rivers, wetlands, and coastal waters to the public (Steinwender et al. 2008; Pratap et al. 2013).

### 1.3 Alignment of Public Perceptions with the Empirical Consensus

Public perceptions of the environment may diverge from those held by scientists, managers, and experts (Bennett 2016). For example, Hofmann et al. (2012) found that the public associated tree canopy with the quality of urban green spaces, while managers associated naturalness with the quality of those spaces. Similarly, Cottet et al. (2013) found that experts place greater value on wetlands with lower water trophic states and floating/submerged vegetation, while the public preferred wetlands with reflective water. Public perceptions of the environment may also differ from the empirical consensus of the environment as noted by Baranzini et al. (2010), Domínguez‐Torreiro & Soliño (2011), Kothencz and Blaschke (2017).

The literature comparing perceptions to the empirical consensus is abundant for some environmental domains but limited for others. For example, comparisons of perceptions and empirical measures of freshwater (Artell et al. 2013; Kataria et al. 2012; Marsh et al. 2011; Steinwender et al. 2008) and coastal waters/beaches (Pendleton et al. 2001; Petrosillo et al. 2007; Quilliam et al. 2019; Rodella and Corbau 2020; Pratap et al. 2013) have received considerable attention. Comparisons of perceptions and empirical measures of the marine environment, marine species, and coastal areas have been undertaken for the UK (Jefferson et al. 2014), Italy (Petrosillo et al. 2007; Rodella and Corbau 2020), and the USA (Pendleton et al. 2001). Conversely, comparing public perceptions with empirical measures of wetlands (Cottet et al. 2013), air quality (Williams and Bird 2003), protected areas (Petrosillo et al. 2007; Rodríguez‐Rodríguez 2012), and forests (Aoshima et al. 2018) has received limited exploration. Moreover, few studies have compared perceptions to empirical measures across more than one environmental domain (e.g. Domínguez‐Torreiro and Soliño 2011; Lui et al. 2021).

The public and experts may also interpret different attributes of environmental domains differently (Liu et al. 2021; Steinwender et al. 2008). For example, Jefferson et al. (2014) found that many people interpret murky water as an indicator of poor marine health even though experts disagree with that assessment. Experts may also prefer different indicators to the public. For example, experts may evaluate coastal waters and beaches according to the integrity of the surrounding landscape, whereas the public may focus more on beach cleanliness and accessibility (Rodella and Corbau 2020). The public may also interpret landscape aesthetics (Aoshima et al. 2018; Kothencz and Blaschke 2017; Steinwender et al. 2008), management intensity (Rodríguez‐Rodríguez, 2012), and nearby infrastructure (Hamel et al. 2021) to infer environmental health rather than considering a comprehensive list of attributes.

### 1.4 Implications for Policy and Management

How perceptions are defined and how well they align with the empirical consensus have implications for policy development (Lui et al. 2021), information provided to the public (Quilliam et al. 2019), environmental education programmes (Jefferson et al. 2014; Petrosillo et al. 2007), resource allocation (Adamowicz et al. 1997), and conservation investment (e.g. Kataria et al. 2012). Environmental management decisions and policy that do not take public perceptions, expectations, and knowledge of the environment into account may run counter to community needs and expectations (Bennett 2016; Pendleton et al. 2001) and may cause conflict between the community and conservation managers (Castillo et al. 2021; Peltzer et al. 2019; Jefferson et al. 2015). This conflict, in turn, reduces trust in and legitimacy of scientists, managers, and experts (Bennett 2016; Lui et al. 2021; Pratap et al. 2013); results in unintended consequences on the allocation of resources and effort by the public (Pendleton et al. 2001); and reduces the ability of the public to make informed decisions about environmental engagement (Pratap et al. 2013; Quilliam et al. 2019). However, disregarding expert opinions and only taking public perceptions into account may also cause inefficient efforts and allocation of resources (Andelmann and Fagan 2000).

If the attributes of the environment that the public evaluates differ from those evaluated by conservation managers, the public may believe environmental management is ineffective (Bennett 2016; Rodríguez‐Rodríguez 2012). For example, Rodella and Corbeau (2020) found that beachgoers associated wide beaches and cleanliness with quality, while conservation efforts focus on reducing beach erosion through breakwaters, groynes, and dune enhancement. As a result, beaches that were wide and clean were considered of higher quality and more favourable for recreation than more naturally or intensively managed beaches.

Perceptions may also influence the public's willingness to engage in the environment and the amount of effort they expend to experience nature. Pendleton et al. (2001) found that while the public visited beaches, they did not enter the water if they had a poor perception of the water regardless of public notices regarding water safety. They also found that the public would drive substantially farther to other beaches if they perceived those beaches to have better water quality. Similarly, Liu et al. (2021) found that the public actively avoided water in Beijing because they viewed it as being low quality despite empirical measures to the contrary.

An emphasis on perceptions may result in misallocation of conservation funding from a science‐needs perspective. For example, while a focus on protecting charismatic species may encourage public engagement in conservation, it may also result in underinvestment in other species of significance to the environment (Sitas et al. 2009; Andelmann and Fagan 2000). For example, environmental valuation studies often compare the empirical state or ‘status quo’ to possible alternatives to measure the public's willingness to pay for conservation efforts. However, if public perceptions of the ‘status quo’ differ from the empirical `status quo’, then valuation measures may be biased (Adamowicz et al. 1997; Ahtiainen et al. 2015; Domínguez‐Torreiro and Soliño 2011; Kataria et al. 2012; Marsh et al. 2011) to wit, Kataria et al. (2012) found that the marginal willingness to pay to improve a stretch of river in Denmark was higher for those who believed the water quality `status quo’ was worse than described compared with those who agreed with the empirical water quality `status quo’. Perceptions of quality of some environments may also change over time (Booth et al. 2022), and not accounting for those changes in perception may bias valuation estimations (McClenachan et al. 2018).

### 1.5 This Study

Comparisons of public perceptions to the empirical consensus are abundant for some environmental domains but limited for others. To address this imbalance in current knowledge, this study identifies where similarities and differences exist within and across the ten environmental domains of air, protected natural areas, native bush and forests, marine environments, coastal waters and beaches, marine plants and animals, terrestrial (land and freshwater) plants and animals, natural environments in towns and cities, wetlands, and rivers and lakes. We describe perceptions as measured in a large‐scale, nationally representative survey from New Zealand with empirical measures based on recent publicly available reports and scientific literature. By synthesising these similarities and differences, we aim to help lay‐people, scientists, and policy makers understand their own perceptual biases. This will enable scientists and other experts to communicate their findings more effectively and assist policy makers and regulators in creating regulations that engage the entire community in environment improvement.

## 2 Materials and Methods

The New Zealand Environmental Perceptions Survey (NZEPS) has quantitatively tracked the national public's perceptions of the environment every 2–3 years since 2000, making it the longest‐running survey of its type in the world. The NZEPS tracks public perceptions of a) the state, b) the quality of management, and c) the natural and anthropogenic pressures on the specified environmental “domains” (e.g. air, rivers and lakes, and marine environments). The 2022 survey was completed by 2,098 respondents. The questionnaire was approved by the Manaaki Whenua – Landcare Research social ethics process (SE#2122/18) and the survey sample is representative across gender, age, ethnicity, education, and region (Booth et al. 2022).

We restricted our analysis to the environmental domains included in the 2022 NZEPS, that is, air; protected natural areas; native bush and forests; marine environments; coastal waters and beaches; marine plants and animals; terrestrial (land and freshwater) plants and animals; natural environments in towns and cities; wetlands; and rivers and lakes. The perceptions of the state and quality of management of these domains were measured on a five‐point Likert scale ranging from ‘very good’ to ‘very bad’ plus a sixth option for ‘don’t know’. The state of the environment to which perceptions are qualitatively compared is drawn from reports and data from central government ministries, departments, and other official sources. Other knowledge sources such as traditional knowledge from Māori (the indigenous people of New Zealand) or multigenerational knowledge from the community could be used for comparison and may be of better quality; however, we chose to use central government sources as this information is widely available and reported to the public.

We qualitatively compare public perceptions and the empirical consensus for several reasons. Foremost, the availability, quality, and spatial and temporal resolution of empirical indicators varies widely across domains. For example, the state of air quality is measured across six biophysical indicators, but no indicator is measured across all 16 regions of New Zealand, and some indicators are only collected in three large cities. In addition, perceptions and empirical indicators are measured on different scales, and indicators for only a few domains can be compared “apples‐to‐apples” to perceptions (e.g., A to E ratings for freshwater quality). Classification of the empirical state as “good” or “bad” in this paper is therefore based on a mixture of i) “good” to “bad” classifications stated in the source reports, ii) impressions of “good” to “bad” stated in the source reports, and iii) qualitative interpretation of trends and available data in the source reports. These impressions are then compared with public perceptions in aggregate to account for variation in an individual's definition of “good” and “bad” state.

## 3 Results: Comparison of Public Perceptions to the Empirical Consensus

### 3.1 Air Domain

Air quality in New Zealand is measured directly by six biophysical indicators. Concentrations of airborne particulate matter (PM) with diameters of less than 10 microns (PM_10_, PM_2.5_), of nitrogen dioxide (NO_2_), sulphur dioxide (SO_2_), and carbon monoxide (CO) have improved across most of New Zealand since 2011, and ground‐level ozone (O3) are consistently below the peak‐season 2021 World Health Organization (WHO) guidelines (Ministry for the Environment & Statistics New Zealand 2021b). However, 76% of PM_10_ monitoring sites, 95% of PM_2.5_ monitoring sites, and 71% of NO_2_ monitoring sites exceeded the 2021 WHO 24 h concentration guidelines at least once between 2017 and 2020 (Ministry for the Environment & Statistics New Zealand 2021b). Indicators are also not measured across all regions. For example, no air quality indicators are measured in Gisborne, PM is not measured in Taranaki, and PM_2.5_ is not measured in five other regions.

**FIGURE 2:**
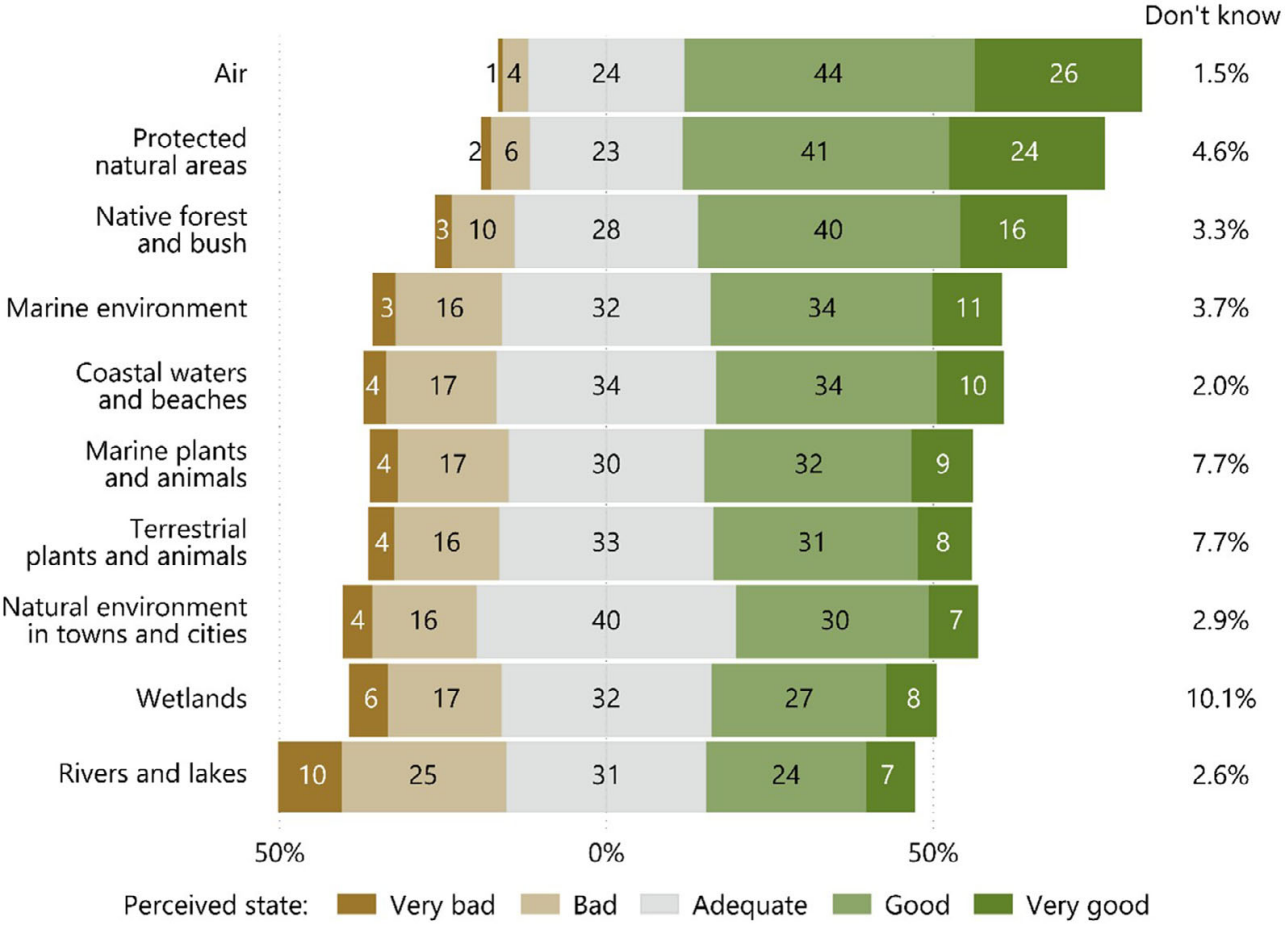
Perceived state of 10 environmental domains from the 2022 NZ Environmental Perceptions Survey.

**FIGURE 3:**
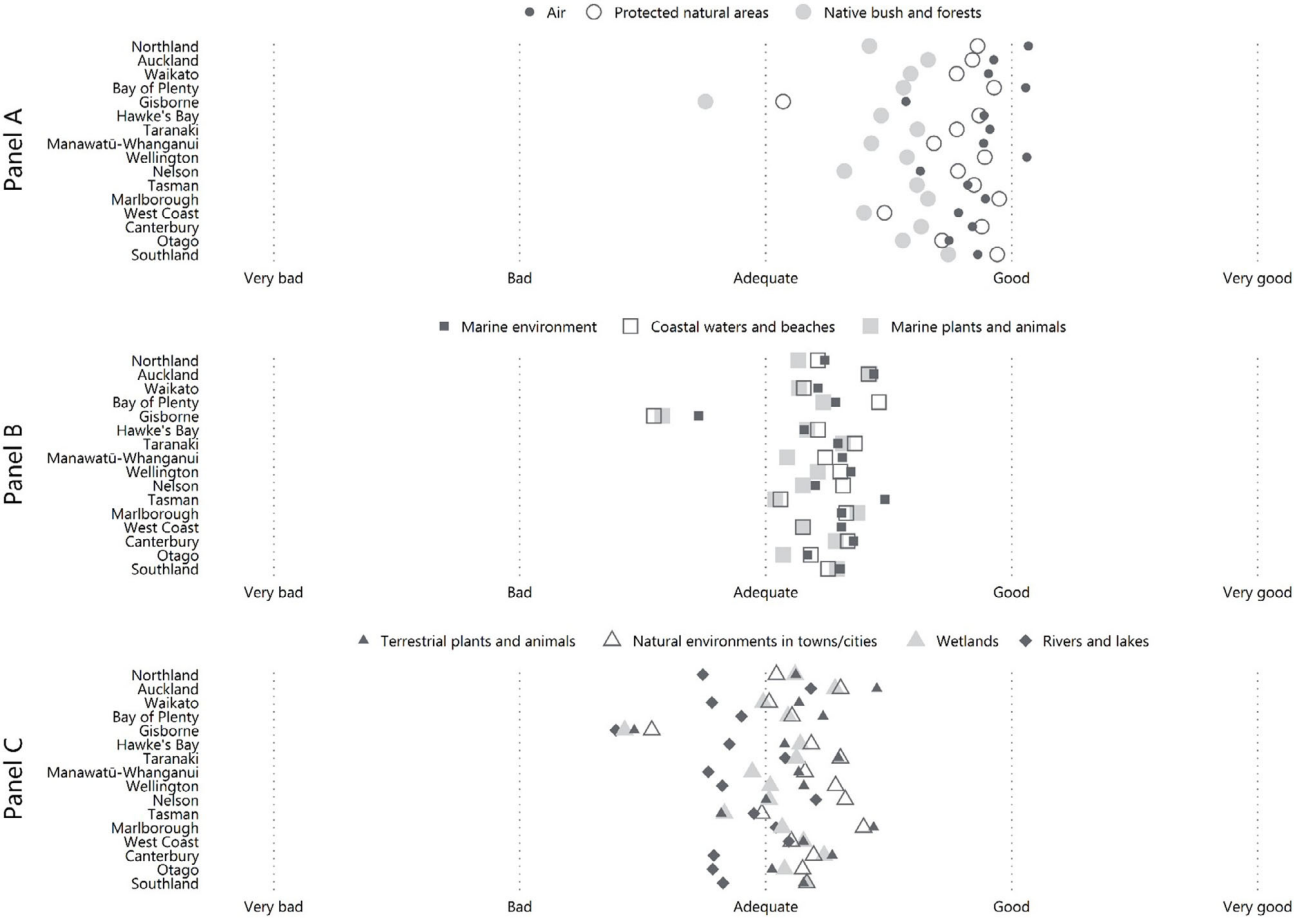
Average perceived state of 10 environment domains from the 2022 NZ Environmental Perceptions Survey by region.

Respondents think the state of New Zealand's air is better than the state of any other environmental domain. Public perceptions of air quality accord with the trends in PM_10_, PM_2.5_, NO_2_, and CO at the national scale, with 70% of respondents thinking air is in a “good” or “very good” state and 5% thinking air is in a “bad” or “very bad” state (Figure 2). However, respondents in Northland, Bay of Plenty, and Wellington think their air quality is good on average (Figure 3, Panel A) despite those in the high‐density parts of Northland and Wellington being exposed to PM_2.5_ concentrations above the WHO's guidelines on a regular basis. That said, no sites in Northland and only 17% of sites in Wellington registered excessive PM_10_ concentrations, suggesting that perceptions may reflect the larger (and more visible) PM_10_ levels than the smaller PM_2.5_. This mixed alignment is similar to results found in the UK (Williams and Bird 2003).

### 3.2 Protected Natural Areas Domain

New Zealand has a network of legally protected areas covering nearly one‐third of the terrestrial land (Landcare Research 2007) including 13 national parks across 8 of its 16 local government regions (Department of Conservation, National parks, n.d) and 44 marine reserves covering 17,430 km^2^ (or 10%) of the economic exclusion zone (Statistics New Zealand 2016). An iwi (Māori tribe) may place a rāhui (a prohibition, ban, or reserve) on certain areas or species or declare that they are tapu (sacred, restricted, or have certain activities relating to them prohibited), but these measures are not always publicly recorded. Assessments of the impact of protected areas on biodiversity (e.g. for wetlands, seabirds) are limited, but they are an area of increasing focus (Department of Conservation 2020; Fisheries New Zealand 2022). However, there have been some positives: for example, trends in the numbers of Māui dolphins (*Cephalorhynchus hectori maui*) showed a potential stabilisation between 2010 and 2016 after the introduction of marine mammalian sanctuaries; the introduction of a marine protected area around Antarctica changed the situation for skates and rays (*Rajidae* family) from being landed catch to being released (Fisheries New Zealand 2022); and the pekapeka / southern lesser short‐tailed bat (*Mystacina tuberculata tuberculata*) is now considered a recovering species as a result of management of population within protected areas (Department of Conservation 2020).

Perceptions of protected natural areas appear to reflect the quantity of terrestrial and marine environments under protection and fauna ‘wins’ connected with that legal protection more than the uncertainty of the empirical state of the flora. Sixty‐five percent of respondents think protected natural areas are in a ‘good’ to ‘very good’ state, 8% of respondent think protected natural areas are in a ‘bad’ to ‘very bad’ state, and fewer than 5% of respondents ‘don’t know’ (Figure 2) Respondents in the Bay of Plenty, Marlborough, and Southland are the most positive about the state of protected natural areas, while respondents in Gisborne are the most negative about the state of protected natural areas (Figure 3, Panel A) despite a similar proportion of land in the Bay of Plenty and Gisborne under legal protection (Landcare Research 2007).

### 3.3 Native Bush and Forests Domain

An estimated 80% of New Zealand's land was covered in native forests prior to human habitation. Presently, only 27% of New Zealand's land area is covered in native forest, an additional 22% is covered in other indigenous ecosystems, and the remaining 51% of land area is covered in urban development, farming, and exotic grasslands and forests (Ministry for the Environment & Statistics New Zealand 2021a). Conversion of indigenous land cover to other land uses such as exotic grassland has continued in the present day; indeed, there was a net loss of indigenous land cover of 17,329 ha between 1996 and 2001, of 31,594 ha between 2001 and 2008, of 26,354 ha between 2008 and 2012, and of 12,869 ha between 2012 and 2018 (Statistics New Zealand 2021a) with variation across the regions. For example, between 2012 and 2018, the West Coast lost a net 1,423 ha of native forest while Manawatū‐Wanganui gained a net 3,395 ha of indigenous forests. That said, Manawatū‐Wanganui also saw a net loss of 4,983 ha of indigenous scrub/scrubland over the same period (Statistics New Zealand 2021a).

Despite trends in losses of indigenous land cover, 56% of respondents think native bush and forests are in “good” to “very good” state (Figure 1). Respondents in Southland rated the state of native bush and forests the highest and respondents in Gisborne rated the state of native bush and forests the lowest, on average, across the country (Figure 2. Panel A). These perceptions contrast with the empirical state of indigenous land cover in these regions; between 2001 and 2018, Southland lost 1,222 ha but Gisborne gained 2,251 ha of indigenous forest cover (Statistics New Zealand 2021a).

### 3.4 Marine Environments Domain

New Zealand's Exclusive Economic Zone (EEZ) is 15 times larger than its land area (Land Information New Zealand 2019). Unfortunately, these extensive deep waters within the EEZ mean high costs to monitoring limit knowledge, and understanding of the condition of marine environments. What knowledge is available shows a general decline over time: sea‐surface temperatures have increased 0.2°C each decade since the 1980's, while ocean acidity has increased 7.1% since the 2000 (Ministry for the Environment & Statistics New Zealand 2019, 2022). Marine environments are under threat from climate change, off‐shore commercial activities such as shipping, fishing, and mining, as well as from nonnative species; for example, between 2010 and 2017, 21 of 49 newly detected nonnative marine species showed signs of becoming established (Ministry for the Environment & Statistics New Zealand 2022).

One‐third of respondents think New Zealand's marine environments are in a “good” state and 11% think its marine environments are in a “very good” state. In comparison, 16% of respondents think marine environments are in a “bad” state and 3% think they are in a “very bad” state (Figure 2). Regionally, respondents in Gisborne, Hawke's Bay, and Otago are the least positive about the state of marine environments, and respondents in Auckland and Nelson are the most positive about the state of their marine environments (Figure 3, Panel B). While perceptions of respondents in Otago are unsurprising given the long‐term ocean acidity trends, perceptions of respondents in Auckland seem more diverged from the empirical state of ocean acidity in their immediate region. However, some indicators of the empirical state are not measured across most of the country so comparison with perceptions is not possible.

### 3.5 Coastal Waters and Beaches Domain

The state of New Zealand's 15,000 km of rocky shore, fjords, inlets, soft beaches, harbours, sounds, and estuaries is generally worse than in open ocean. Plastic particulates have been found in measurable quantities across New Zealand's beaches since 1972 and now comprise most of the litter on New Zealand beaches (Gregory 1978; Ministry for the Environment & Statistics New Zealand 2019). Increasing extreme wave events (>8 m) coupled with sea‐level rise and increasing sea temperatures exacerbate erosion and degradation of the coastline (Ministry for the Environment & Statistics New Zealand 2019). Between 1977 and 2013, total nitrogen and nitrate‐nitrites loads entering New Zealand's coastal waters increased 74% and 159% while total phosphorus and dissolved reactive phosphorus loads entering New Zealand's coastal waters increased 48% and 18% (Ministry for the Environment & Statistics New Zealand 2022). Between 2011 and 2020, declining trends in quality are more common than improving trends in monitored estuaries for ammoniacal nitrogen, faecal coliform, and dissolved oxygen. However, more than 60% of monitored estuaries showed an improving trend in nitrate–nitrite nitrogen, total phosphorus, and suspended solids over the same period (Statistics New Zealand 2022a); and more recently, heavy metal concentrations were below levels expected to affect bottom‐dwelling species (Ministry for the Environment & Statistics New Zealand 2020).

Alignment of perceptions and the empirical state of coastal waters and beaches is mixed depending on the region. In aggregate, 54% of respondents think coastal waters and beaches are in an “adequate” to “good” state while 21% of respondents think coastal areas are in a “very bad” to “bad” state (Figure 2). This latter group is primarily located in Gisborne, Nelson, and the West Coast (Figure 3, Panel B). However, respondents in Auckland and Bay of Plenty are the most positive about the state of their coastal waters even as Auckland regularly measures highly acidic waters and worsening trends in chl‐a, and Bay of Plenty experiences regular extreme wave events (>8 m) (Ministry for the Environment & Statistics New Zealand 2019). These results suggest that respondents may consider different indicators of coastal and beach health than scientists. However, as with indictors for marine environments, many coastal water indicators are measured near high population centres (e.g., Auckland, Bay of Plenty, Canterbury), which may not accurately reflect the conditions that other respondents are experiencing.

### 3.6 Marine Plants and Animals Domain

An estimated 38% of known marine plants and animals are endemic. ≈13,000 marine species have yet to be discovered, but that estimate is conservative given the broad geomorphology of New Zealand's EEZ including high seismic activity (Fisheries New Zealand 2022). Eighty‐four percent of fish stocks that are managed under the quota system and are regularly assessed are in good state, while 16% are considered overfished. However, 32% of fish stocks managed under the quota system have not been scientifically assessed, and by‐catch species are not usually recorded (Ministry for the Environment & Statistics New Zealand 2019). Sixteen percent of marine mammals, 31% of seabirds, and 47% of shorebirds that have been scientifically assessed were considered threatened by extinction (Ministry for the Environment & Statistics New Zealand 2022). Since 1997, phytoplankton abundance has declined in the northern waters of the North Island and off the west coast of the South Island but has increased elsewhere. Sewage and stormwater run‐off, human‐made plastics, pharmaceutics and chemicals, and climate change also affect marine plants and animals. (Ministry for the Environment & Statistics New Zealand 2019).

Alignment of perceptions and the empirical state of marine plants and animal is mixed. Forty‐one percent of respondents think marine plants and animals are in a ‘good’ to ‘very good’ state reflecting the relatively positive state of fish stocks under the quota management system. However, 21% think marine plants and animals are in a ‘bad’ to ‘very bad’ state and 7.7% of respondents ‘don’t know’ the state of marine plants and animals (Figure 2), reflecting the high degree of uncertainty around un‐assessed fish species and the poor state of marine mammals. There is also large variation in uncertainty of perceived state across regions: 0% of respondents in Tasman and West Coast ‘don’t know’ the state while 14% of respondents in Gisborne and Marlborough ‘don’t know’ the state. Additionally, perceptions of state held by respondents in Auckland are more positive while those in Gisborne are more negative than respondents in other regions (Figure 3, Panel B).

### 3.7 Terrestrial (land and Freshwater) Plants and Animals Domain

Twenty‐seven percent of New Zealand's original prehuman land cover remains; 81 plant and animal species have become extinct since first human contact, including 62 bird species (Robertson et al. 2021). In addition, 43% of native freshwater fish, 12% of native freshwater and 8% of native terrestrial invertebrates, 15% of vascular plants, 30% of native terrestrial birds, and 40% of native reptiles are threatened with extinction (Ministry for the Environment & Statistics New Zealand 2020, 2022). There is insufficient data on 37% of terrestrial flora and fauna—mainly fungi, lichen, and insects—to assess their current state (Department of Conservation 2020). Among the 71 ecosystems identified as “rare”, 45 are threatened with collapse (Ministry for the Environment & Statistics New Zealand 2022). However, there are bright spots within this domain. One‐third of terrestrial species are classified as not threatened (Department of Conservation 2020), and there have been a few very public ecological success storeys. For example, the once‐thought‐extinct Takahē was rediscovered in 1948 and now has a population exceeding 400 (Department of Conservation, Takahē, n.d.).

Similar to the proportion of terrestrial species at‐risk of extinction, not threatened by extinction, or with an uncertain condition, respondents’ perceptions of the state of terrestrial plants and animals is also divided: 39% of respondents think this domain is in a “good” to “very good” state, 33% think it is in an “adequate” state, and 20% think it is in a “bad” to “very bad” state (Figure 2). This latter group is driven by respondents in Gisborne and Nelson (Figure 3, Panel C). Respondents in Auckland and Marlborough are the most positive about the state of terrestrial plants and animals.

### 3.8 Natural Environment in Towns and Cities Domain

Eighty‐seven percent of New Zealand's population currently resides in urban areas, representing just under 1% (240,000 ha) of the country's land (Statistics New Zealand 2017). Access to green spaces in urban centres is unevenly distributed. For example, 47% of Auckland, 45% of Hamilton, and 65% of Wellington is considered green space, but <20% of Auckland, <20% of Hamilton, and 41% of Wellington is considered public green space. The remaining green areas in these areas is on private land (e.g. lawns) or along transport corridors (Parliamentary Commissioner for the Environment 2023). Modelling indicates that nitrate‐nitrogen levels are 23 times higher, *E. coli* levels are 26 times higher, dissolved reactive phosphorus levels at four times higher, and turbidity levels are three times higher in rivers in catchments dominated by urban land cover compared to rivers in indigenous land cover. However, turbidity trends have improved at 72% of monitored urban sites, nitrate‐nitrogen trends have improved at 70% of urban sites, dissolved reactive phosphorous trends have improved at 64% of urban sites, and ammoniacal nitrogen trends have improved at 55% of urban sites in recent years (Ministry for the Environment & Statistics New Zealand 2020).

Respondents think natural environments in towns and cities are generally adequate, with 37% of respondents saying natural environments are in a “good” to “very good” state and 20% of respondents saying natural environments are in a “bad” to “very bad” state (Figure 2). In Gisborne, respondents consider natural environments in developed areas to be in a “bad” or “very bad” state, on average (Figure 3, Panel C). In contrast, respondents in Marlborough are the most positive about the state of the natural environments in developed areas followed by respondents in Auckland, Taranaki, Tasman, and Wellington. Unfortunately, most information on green spaces and urban waterways is from the largest cities—Auckland, Wellington, Hamilton, and Christchurch—so comparison of the empirical state to perceptions held by respondents in other regions is not feasible.

### 3.9 Wetlands Domain

Less than 10% of the prehuman settlement extent of wetlands remain in New Zealand today (Ministry for the Environment & Statistics New Zealand 2020; Dymond et al. 2021) and 60% of these remaining wetlands are estimated to be in a moderately to severely degraded state (Ausseil et al. 2011). However, publicly available studies on the detailed condition of wetlands including trajectories of degradation and restoration, are limited to a handful of wetlands, for example, Moanatuatua, Kopuatai, and Torehape peat bogs (Clarkson and Bartlam 2017; Watts et al. 2020) and lowland Whangamarino wetland (Blyth et al. 2013). As such, the state of wetlands across New Zealand remains poorly monitored and poorly understood (Ministry for the Environment & Statistics New Zealand 2020).

While respondents’ perceptions do not correspond with the empirical state of wetlands in aggregate, the distribution of perceptions perhaps reflect the differences between what is empirical documented and what is not empirical documented about the state of wetlands in New Zealand. For example, one‐third of respondents think wetlands are in a ‘good’ or ‘very good’ state, and 23% think wetlands are in a ‘bad’ or ‘very bad’ state (Figure 2). However, 10% of respondents ‘don’t know’ the state of wetlands, the greatest uncertainty level recorded for any environmental domain. Regionally, there is also little alignment between perceptions and change in extent of freshwater or saline wetlands. Although Auckland has lost 50.3 ha of saline wetlands and Canterbury has lost 8.7 ha of saline and 85.1 ha of freshwater wetlands since 2008 (Statistics New Zealand 2021b), respondents in Auckland and Canterbury have the most positive view of the state of wetlands. In contrast, Nelson has lost 9.4 ha of saline wetlands and Gisborne has lost 4.9 ha of saline and 25.3 ha of freshwater wetlands since 2008 (Statistics New Zealand 2021b), Respondents in Nelson and Gisborne have the worst opinion of the state of wetlands (Figure 3, Panel C). Southland lost 1655.4 ha of freshwater wetlands between 2008 and 2018 (Statistics New Zealand 2021b) and respondents in Southland are the most uncertain about the state of their wetlands (15% of respondents ‘don’t know’ the state of wetlands).

### 3.10 Rivers and Lakes Domain

Across the country, 64% of river length (fourth order or higher rivers only) have phosphorus concentrations in excess of natural levels, 69% have nitrogen concentrations in excess of natural levels, 37% of river length are impaired by turbidity, and 9% have significantly impaired visual clarity (Ministry for the Environment & Statistics New Zealand 2022). Only 7% of rivers have “pristine” macroinvertebrate community index scores (Ministry for the Environment & Statistics New Zealand 2022). Nearly all rivers in catchments dominated by urban land cover, exotic forest land cover, and pastoral land cover exceeded at least one nationally set nutrient or turbidity limit between 2013 and 2017. In addition, 94% of the river length in catchments with urban land cover and 76% of it in catchments with pastoral land cover are considered unsafe for swimming (Ministry for the Environment & Statistics New Zealand 2020). Indeed, a *Campylobacter* infection risk greater than 3% was found at 45% of modelled rivers nationwide (The National Policy Statement for Freshwater Management classifies a river or lake with an infection risk greater than 3% of *Campylobacter* as “poor” for human contact). Between 2016 and 2020, median *E. coli* concentrations exceeded maximum safe concentrations at 15% of modelled rivers (Statistics New Zealand 2022b).

Of the 7.7% of lakes larger than 1 ha with available quality data, 34% were in excellent or high ecological state, 31% were in moderate ecological state, and 36% were in poor ecological state or lacked any submerged plants (Ministry for the Environment & Statistics New Zealand 2020). Across 101 monitored lakes, 11.9% were microtrophic or oligotrophic, and 62.4% were eutrophic or supertrophic (Statistics New Zealand 2022a). The modelled trophic lake index (for lakes larger than 1 ha) was “poor” or “very poor” for over two‐thirds of lakes in catchments dominated by urban land cover, pastoral land cover, and exotic forest land cover. This compared with it being “poor” or “very poor” in 19% of lakes in catchments dominated by native land cover (Ministry for the Environment & Statistics New Zealand 2020).

Respondents rank the state of rivers and lakes lower than the state of any other environmental domain, largely reflecting the poorer empirical state discussed above. However, a similar proportion of respondents think rivers and lakes are in a “good” to “very good” state (31%) as think they are in an “adequate” (31%) or “bad” to “very bad” state (35%; Figure 2). Respondents in Auckland are the most positive about the state of freshwater (Figure 3, Panel C) despite the empirical state of freshwater resources in urban areas being particularly poor. While respondents in Tasman are also very positive about the state of their rivers and lakes, there is limited data available in that region to compare to the empirical state of freshwater in that region.

## 4 Discussion

Effective environmental policy requires balancing public expectations (based on public perceptions) with desired conservation outcomes (informed by the empirical consensus). Focusing on the ten environmental domains of air, protected natural areas, native bush and forests, marine environments, coastal waters and beaches, marine plants and animals, terrestrial (land and freshwater) plants and animals, natural environments in towns and cities, wetlands, and rivers and lakes in New Zealand, we identified where similarities and differences between public perceptions and the empirical consensus exist. In this Discussion, we briefly review where alignment and misalignment occur, discuss where these differences may originate, describe how the findings may inform environmental communication and policy, and outline opportunities for future research.

Misalignments between public perception and empirical consensus have been documented in other disciplines. For instance, in nutrition science, consumers often misjudge the healthiness of food products based on simplified labels or packaging cues, leading to choices that diverge from nutritional guidelines (Thomas et al. 2021; Machín et al. 2020). In climate science, despite overwhelming consensus among experts, public belief in anthropogenic climate change remains fragmented, shaped by political identity and media narratives (Bliuc et al. 2015; Cologna et al. 2024). These parallels underscore the broader challenge of aligning public understanding with scientific evidence across domains.

### 4.1 Alignment and Misalignment

The public believed that the state of New Zealand's air is better than the state of any other environmental domain and that the state of rivers and lakes was the poorest. These public perceptions are both in accord with the available empirical measures. For the other domains, public perceptions were varied, and the overall state was generally seen as better than what the empirical consensus suggested.

### 4.2 Interpretation

Misalignment between public perception and the empirical consensus reflects a complex interplay between the factors such as public awareness, visibility, familiarity of environmental issues, past experience, and media influence. For example, perceptions of air quality are influenced by sensory input and significant but low‐frequency events, so air quality improvements are often easily noticeable. In a similar vein, the degradation of rivers and lakes has received a high level of attention both in the media and in the actions taken by policymakers in recent years (Ministry for the Environment 2023), leading to a concurrence in environmental perceptions and the empirical consensus. In the weeks preceding the survey, New Zealand's East Cape experienced severe flooding. While these events are related to climate rather than environment per se, they could potentially influence respondents’ perceptions of environmental domains.

The general public optimism about other environmental domains could stem from a lack of detailed knowledge or exposure to the nuanced challenges facing these domains. For example, low familiarity and public uncertainty about the condition of marine domains and marine flora and fauna may reflect the low quality and quantity of accessible knowledge (Jefferson et al. 2014; Pendleton et al. 2001) as well as gaps in the empirical knowledge of marine areas (Fisheries New Zealand 2022; Ministry for the Environment & Statistics New Zealand 2019).

In some cases, the public and experts interpret different attributes of environmental domains differently (Liu et al. 2021; Steinwender et al. 2008). For example, people are more confident in their perceptions of freshwater and coastal waters because they can use sight and smell to assess quality. They may interpret a “tidy” visual aesthetic (e.g. clear reflective water with little submerged vegetation) of lakes, rivers, wetlands, and coastal beaches as indicators of high quality, whereas experts often look for submerged vegetation in wetlands and lakes and natural marine debris on coastal beaches as environmental indicators.

Perceptions and the empirical consensus may also diverge because of biases in empirical knowledge and environmental indicators. Data limitations, complexity of ecosystems, and funding priorities influence which environments are studied and how the condition of those environments are communicated (Layke et al. 2012). For example, freshwater and soil erosion are often subject to government regulation, resulting in better data as well as indicators to track trends over time (Layke 2009; Layke et al. 2012). Indeed, the type and quality of empirical measures available in New Zealand directly influenced our own methodological rationale and interpretation of alignment/misalignment of perceptions with the empirical consensus. For some environmental domains, significant gaps in knowledge meant that we relied on qualitative analysis.

Beyond perceptual biases, it is also important to consider how the communication of ecological indicators may reflect the values and assumptions of the communicators (Harmsworth et al. 2011). For example, the Millennium Ecosystem Assessment's (MEA) conceptualisation of ecosystems as stocks and flows alienated people that did not view nature through those lenses (Diaz et al. 2018). Consequently, research based on the MEA framework has remained narrow in scope, limiting its relevance and uptake among lay audiences and policymakers who do not share those foundational assumptions. Integrating differing value systems and world views into the design of ecological indicators could help reduce bias in empirical measures and improve their alignment with public perceptions (Medin and Bang 2014a).

### 4.3 Implications

Our results highlight the need for better information and transparent communication from environmental experts to bridge the gap between public perception and empirical consensus. Information programmes and communication of environmental quality may help counteract divergent perceptions if they are designed with these perceptions in mind. For example, Pratap et al. (2013) found that while flags and signs at the beach, indicated suitability of water for swimming, only a handful of people actively looked for that information once they arrived at the beach, and only one‐third of the people actually understood the information. Instead, the public relied on their individual perceptions of clean water when deciding whether to swim. Thus, communicating environmental quality through other sources such as the local news, radio, or weather reports could better change beachgoer behaviour.

### 4.4 Limitations and Future Research

Biases and limitations exist within the current study and stem from at least three sources. First, there is considerably more information available on the freshwater and coastal domains than other domains. As a result, our understanding of factors that influence alignment/misalignment is skewed toward those domains.

Second, our interpretation of alignment / misalignment between public perceptions and the empirical measures is influenced by the type and quality of available empirical measures. For example, there are known knowledge gaps in empirical measures for quality of the marine environment and wetlands—gaps that impact both this study's interpretation as well as the general public's understanding of those domains.

Third, it is important to recognise that the design and communication of ecological indicators may carry interpretive biases shaped by the worldviews and values of those who create them. That is, the empirical consensus is created by people who—like anyone else—are influenced by past experience, cultural traits and values, and so on.

For future research, we advocate expanding empirical assessments in underrepresented domains such as wetlands, protected areas, and native forests. We also advocate for research that more fully explores how different cultural and social contexts influence both environmental perceptions and how the empirical consensus can reflect a wider range of values and knowledge systems—including Indigenous and local perspectives—to improve their legitimacy and resonance with diverse communities. By addressing these gaps, future studies can contribute to more inclusive and effective environmental communication and policy development.

## 5 Conclusions

This study addressed the imbalance in current knowledge by examining perceptions across ten key environmental domains: air; protected natural areas; native bush and forests; marine environments; coastal waters and beaches; marine plants and animals; terrestrial (land and freshwater) plants and animals; natural environments in towns and cities; wetlands; and rivers and lakes. By highlighting the similarities and differences in how laypeople, scientists, and experts view these domains, we have provided an insight into the perceptual landscape. However, our findings also underscore the need for further research in several areas. Specifically, more systematic comparisons of perceptions across different groups and ecosystems and the factor(s) that influence alignment or divergence of those perceptions, such as media, salience, and familiarity with environmental issues. Little has been done into the influence of structural and social biases on perceptions and communication of environmental management. Additionally, there is a need to investigate the effectiveness of different communication strategies in bridging the gap between public perceptions and empirical assessments across different ecosystems. By addressing these gaps, future research can further enhance the development of effective environmental communications and policies that engage the entire community in environmental improvement.

## Funding

This study was supported by Ministry of Business, Innovation and Employment.

## Conflicts of Interest

The authors declare no conflicts of interest.
